# Supplementary material for: Human cytomegalovirus UL78 is a nuclear-localized GPCR necessary for efficient reactivation from latent infection in CD34+ hematopoietic progenitor cells
Source: J Virol. 2025 Oct 8;99(11):e01402-25. doi: 10.1128/jvi.01402-25 (PMC12645970; doi:10.1128/jvi.01402-25)
Supplement: Supplemental material — Figures S1 to S7 and Table S1. [file jvi.01402-25-s0007.docx]

**Supplemental Material: Human Cytomegalovirus UL78 is a Nuclear-Localized GPCR Necessary for Efficient Reactivation from Latent Infection in CD34^+^ Hematopoietic Progenitor Cells**

Samuel Medica^a*^, Nicole L. Diggins^a*^, Michael Denton^a^, Rebekah L. Turner^a^, Lydia J. Pung^a^, Adam T. Mayo^a^, Olivia Kramer-Hansen^b^, Jennifer Mitchell^a^, Luke Slind^a^, Linh K. Nguyen^a^, Teresa A. Beechwood^a^, Gauthami Sulgey^a^, Craig N. Kreklywich^a^, Daniel Malouli^a^, Mette M. Rosenkilde^b^, Patrizia Caposio^a^, Daniel N. Streblow^a^, & Meaghan H. Hancock^a^

^a^ Vaccine & Gene Therapy Institute, Oregon Health & Science University, Beaverton, Oregon, USA

^b^ Department of Biomedical Sciences Molecular Pharmacology, University of Copenhagen, Copenhagen, Denmark

* These authors contributed equally to this work

**Running Title: HCMV UL78 is Required for Efficient Viral Reactivation**

^#^Address correspondence to Meaghan H. Hancock, [hancocme@ohsu.edu](mailto:hancocme@ohsu.edu)

**Supporting Information Legends**

**
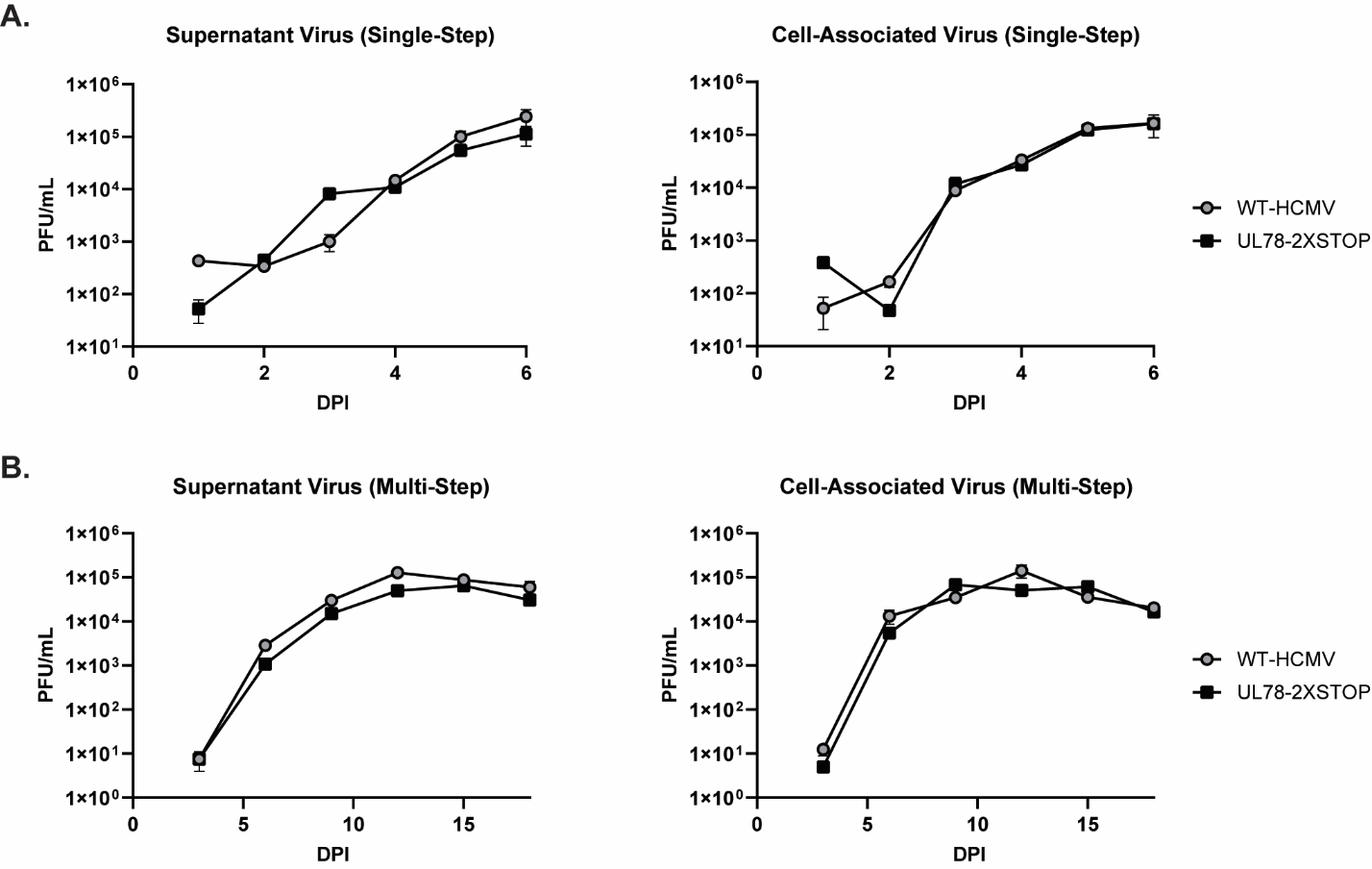
**

**Supplemental Figure 1: HCMV UL78 is Not Required for Lytic Replication.** NHDF cells were infected with the indicated viruses at a MOI of **(A)** 3 or **(B)** 0.01. Supernatant and cell-associated virus were harvested at the indicated timepoints post-infection and titered using confluent monolayers of NHDFs. Error bars represent the standard error of the mean between biological triplicates.


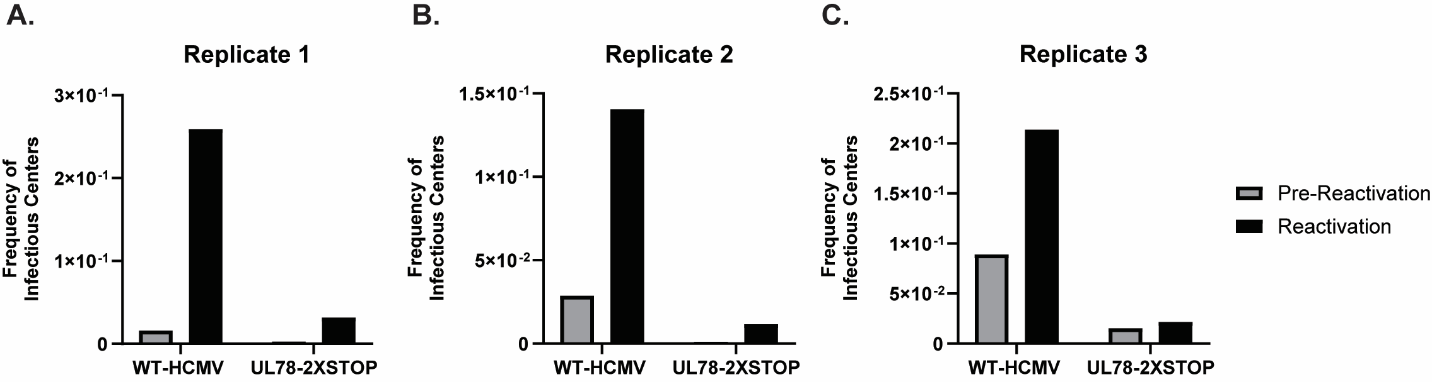


**Supplemental Figure 2: Replicate Experiments for HCMV UL78-2XSTOP Latency Assays.** hESC-derived CD34^+^ HPCs were infected with either TB40/E-GFP (WT-HCMV) or TB40/E-GFP-UL78-2XSTOP (UL78-2XSTOP) at a MOI of 2 for 48 hrs. Cells were FACS isolated for viable CD34^+^/GFP^+^ HPCs and were cultured above a murine stromal cell support layer for 12 days to establish latent infection. **(A – C)** At 14-dpi, half of the cells were treated with reactivation cocktail and plated onto a fibroblast monolayer (reactivation). Reactivation was assessed by the frequency of infectious centers as determined via ELDA and compared to lysed cells (pre-reactivation) at 3 weeks post-plating.


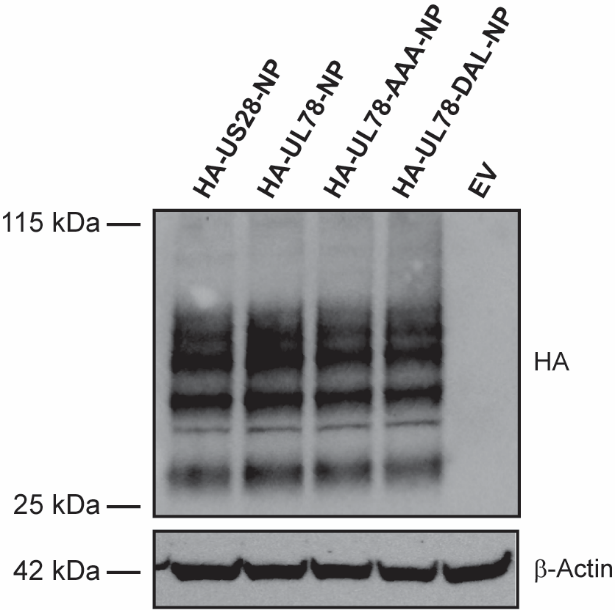


**Supplemental Figure 3: Natural Peptide Tagged Constructs are Efficiently Expressed.** HEK-293 cells were transfected with the indicated constructs. Expression was confirmed via immunoblot using the indicated antibodies on whole lysates harvested 48 hrs post-transfection. Representative blot shown from triplicate experiments.

**
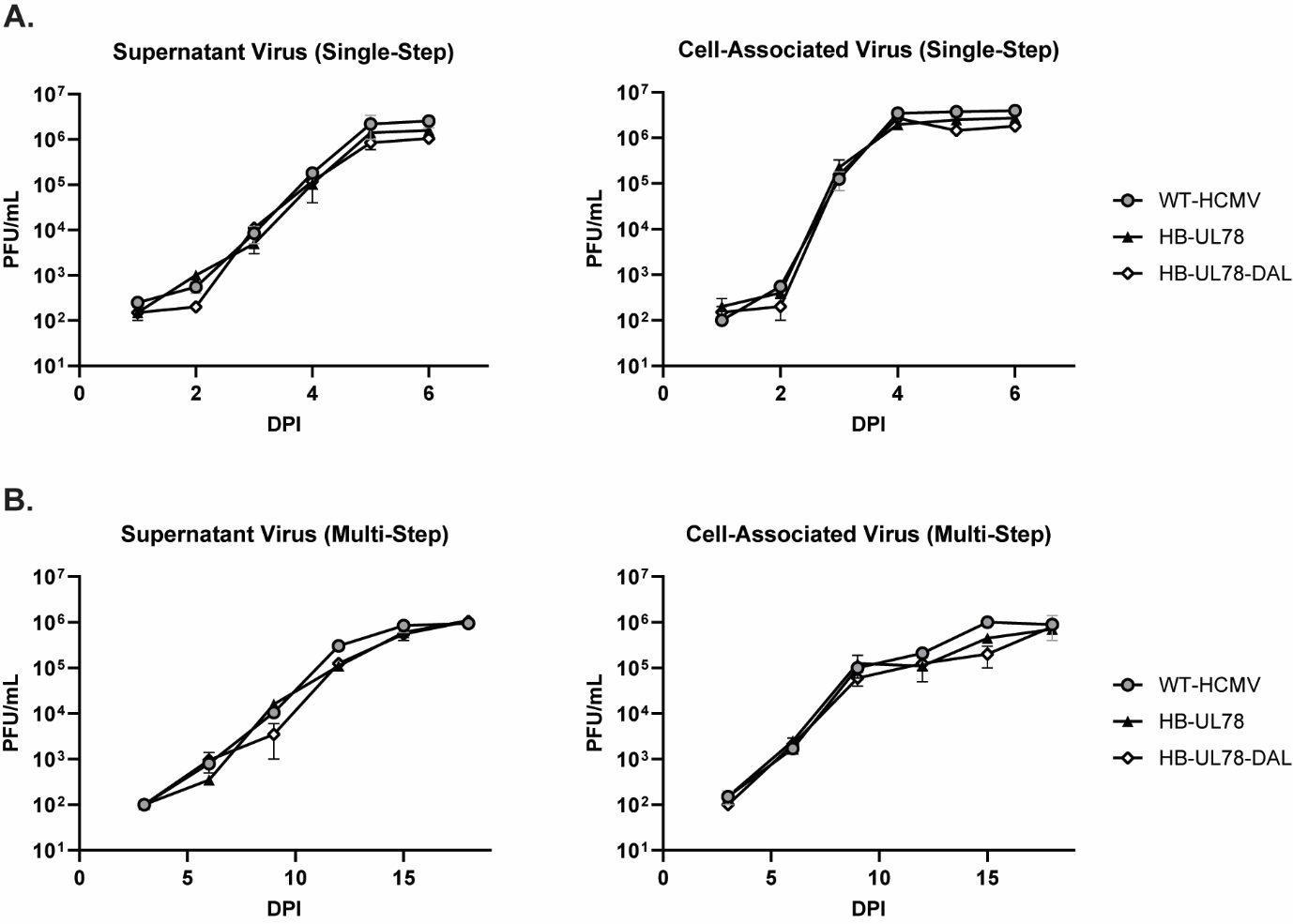
**

**Supplemental Figure 4: HCMV UL78 G-Protein Coupling is Not Required for Lytic Replication.** NHDF cells were infected with the indicated viruses at a MOI of **(A)** 3 or **(B)** 0.01. Supernatant and cell-associated virus were harvested at the indicated timepoints post-infection and titered using confluent monolayers of NHDFs. Error bars represent the standard error of the mean between biological triplicates.


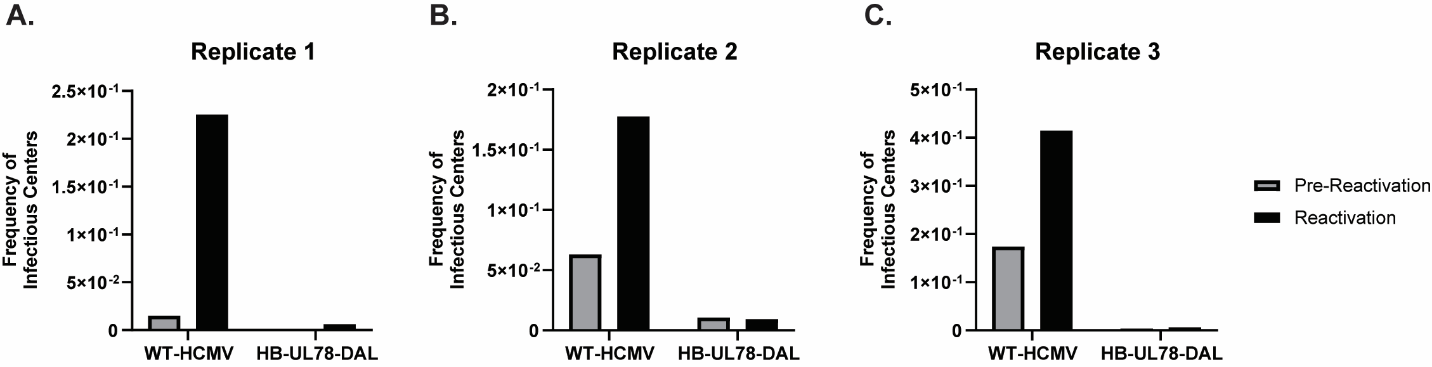


**Supplemental Figure 5: Replicate Experiments for HCMV HB-UL78-DAL Latency Assays.** hESC-derived CD34^+^ HPCs were infected with either TB40/E-GFP (WT-HCMV) or TB40/E-GFP-HB-UL78-DAL (HB-UL78-DAL) at a MOI of 2 for 48 hours. Cells were FACS isolated for viable CD34^+^/GFP^+^ HPCs and were cultured above a murine stromal cell support layer for 12 days to establish latent infection. **(A – C)** At 14 dpi, half of the cells were treated with reactivation cocktail and plated onto a fibroblast monolayer (reactivation). Reactivation was assessed by the frequency of infectious centers as determined via ELDA and compared to lysed cells (pre-reactivation) at 3 weeks post-plating.


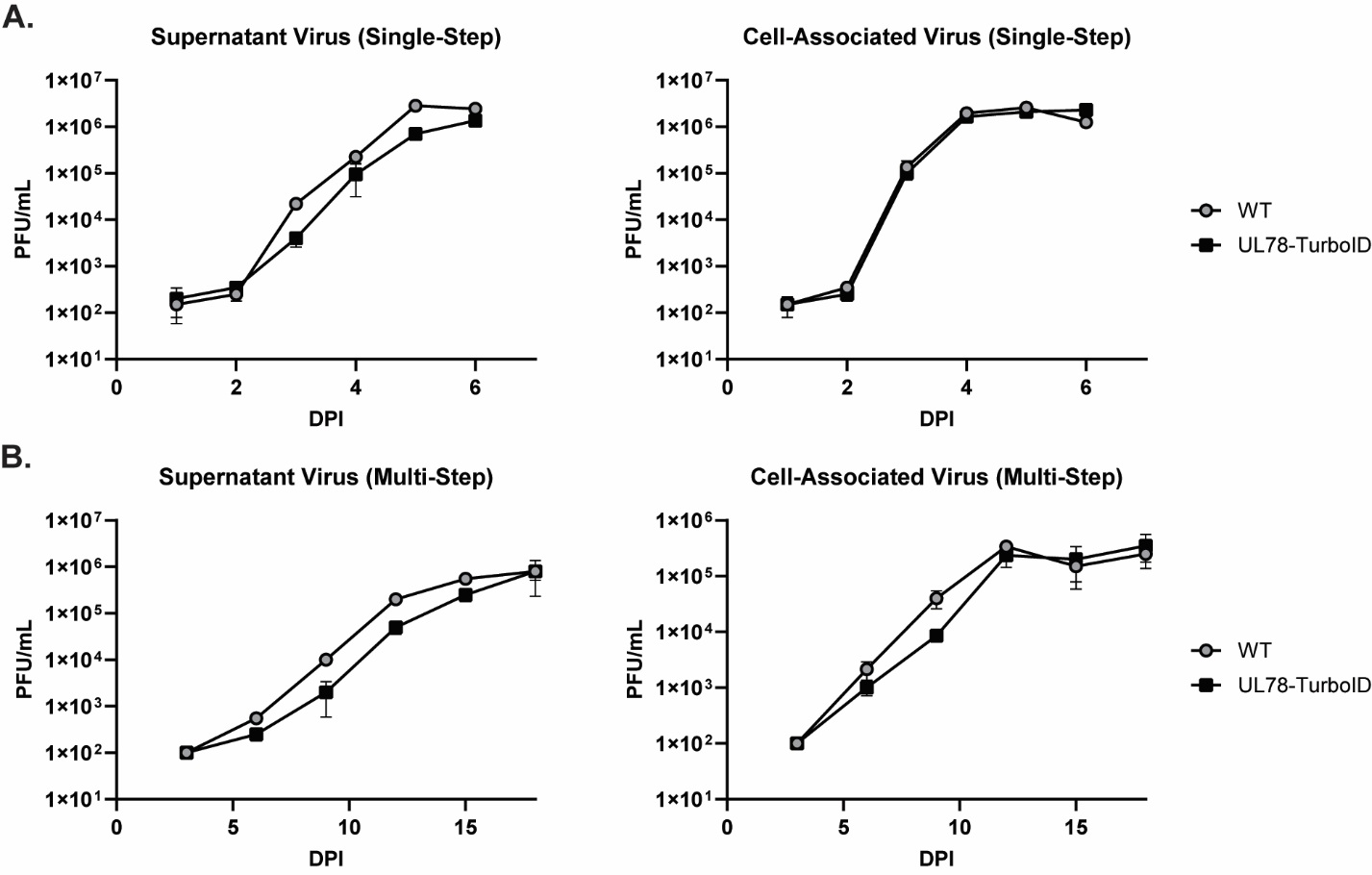


**Supplemental Figure 6: Growth Analysis of Recombinant UL78-TurboID Virus.** NHDF cells were infected with the indicated viruses at a MOI of **(A)** 3 or **(B)** 0.01. Supernatant and cell-associated virus were harvested at the indicated timepoints post-infection and titered using confluent monolayers of NHDFs. Error bars represent the standard error of the mean between biological triplicates.


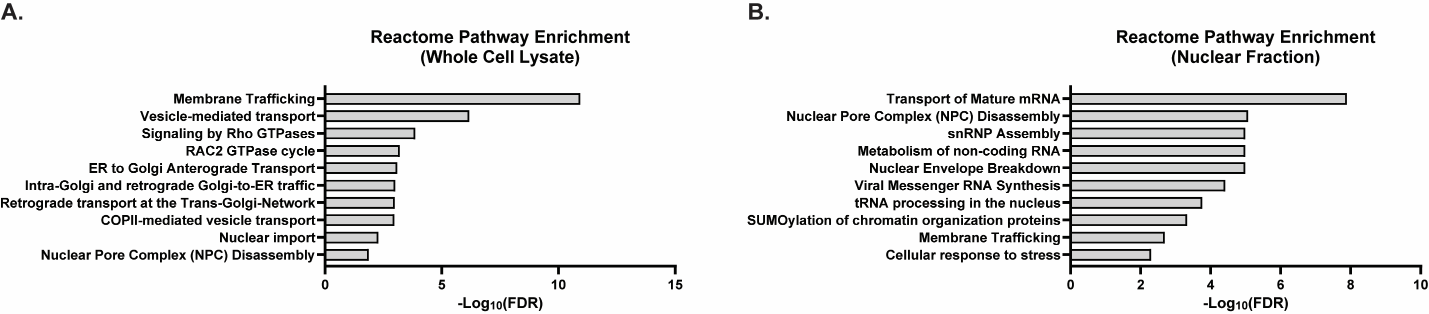


**Supplemental Figure 7: Over-Representation Analysis of Candidate UL78 Interaction Partners.** Reactome over-representation analysis on proteins identified through proximity-dependent labeling experiments in NDHF cells utilizing **(A)** whole cell lysates or **(B)** nuclear extracts.

| **Target** | **Designation** | **Sequence** |
| --- | --- | --- |
| HA-UL78-Natural Peptide | Forward | GCCGCCACCATGTACCCGTACGACGTCCCAGACTACGCCTCCCCTTCTGTGGAGGAGACTACC |
|  | Reverse | GGATTCGCTCACACAGCCGCCAGCCGGTCACACCTAATGCCGTCACCGTTGCGTCGG |
| HiBiT-UL78 | Forward | GAATTCGCCGCCACCATGGTGAGCGGCTGGCGGCTGTTCAAGAAGATTAGCTCCCCTTCTGTGGAGG |
|  | Reverse | AAGCTTTCATCATAATGCCGTCACCGTTGCGTCGGACGCGACGGTGTTTTCGCCGTCGG |
| HA-US28-Natural Peptide | Forward | GTCGCGTCTCACAAATTATACCGGGCGGCATGAAGACGATCATCGC |
|  | Reverse | GCGATGATCGTCTTCATGCCGCCCGGTATAATTTGTGAGACGCGAC |
| HA-UL78-DAL-Natural Peptide | Forward | TCCTCTTTCTGATACTGGATGCTCTGTCGGCCATATCTTACG |
|  | Reverse | CGTAAGATATGGCCGACAGAGCATCCAGTATCAGAAAGAGGA |
| HA-UL78-AAA-Natural Peptide | Forward | TTTCCTCTTTCTGATACTGGCGGCCGCCTCGGCCATATCTTACGGCCG |
|  | Reverse | CGGCCGTAAGATATGGCCGAGGCGGCCGCCAGTATCAGAAAGAGGAAA |

**Supplemental Table 1:** Primers used for generating plasmid constructs. Sequences are shown from the 5’ to 3’ orientation.
